# Establishment of rat model for aspiration pneumonia and potential mechanisms

**DOI:** 10.1002/ame2.12566

**Published:** 2025-03-20

**Authors:** Hanbing Hu, Junfeng Chen, Yiru Shao, Yuedong Tang, Yu Dun, Obulkasim Memet, Xuanrong Bao, Jie Shen

**Affiliations:** ^1^ Center of Emergency and Critical Medicine Jinshan Hospital of Fudan University Shanghai China; ^2^ Research Center for Chemical Injury Emergency and Critical Medicine of Fudan University Shanghai China; ^3^ Key Laboratory of Chemical Injury Emergency and Critical Medicine of Shanghai Municipal Health Commission Shanghai China

**Keywords:** acute lung injury, aspiration, disease models, pneumonia

## Abstract

**Background:**

Aspiration pneumonia is a severe health concern, particularly for ICU patients with impaired airway defenses. Current animal models fail to fully replicate the condition, focusing solely on chemical lung injury from gastric acid while neglecting pathogen‐induced inflammation. This gap hinders research on pathogenesis and treatment, creating an urgent need for a clinically relevant model. This study aimed to develop an improved rat model of aspiration pneumonia by combining hydrochloric acid (HCl) and lipopolysaccharide (LPS) administration.

**Methods:**

Specific pathogen‐free Sprague Dawley rats underwent intratracheal instillation of HCl and LPS. Techniques included rat weight measurement, tracheal intubation, pulmonary function monitoring, lung tissue sampling with HE staining and scoring, bronchoalveolar lavage fluid (BALF) sampling, protein and inflammatory cytokine analysis via BCA and ELISA, BALF pH determination, Evans Blue dye assessment, blood gas analysis, FITC‐dextran leakage, Western blotting, electron microscopy, survival analysis, and transcriptome sequencing with bioinformatics. Statistical analysis was performed using GraphPad Prism.

**Results:**

The optimal model involved instillation of 1.5 μL/g.wt HCl (pH = 1) followed by 20 μg/g.wt LPS after 1 h. This model reproduced acute lung injury, including tissue damage, pulmonary microvascular dysfunction, inflammatory responses, hypoxemia, and impaired pulmonary ventilation, with recovery observed at 72 h. PANoptosis was confirmed, characterized by increased markers. Concentration‐dependent effects of HCl and LPS on lung damage were identified, alongside cytokine elevation and microvascular dysfunction.

**Conclusions:**

This optimized model closely mimics clinical aspiration pneumonia, providing a valuable tool for studying pathophysiology and therapeutic strategies.

## Graphical abstract

Aspiration pneumonia was induced in Sprague Dawley rats by intratracheal instillation of hydrochloric acid (HCl, 1.5 μL/g.wt, pH = 1) followed by lipopolysaccharide (LPS, 20 μg/g.wt) after 1 h. The model reproduced hallmark features of acute lung injury, including histological lung damage, microvascular dysfunction, inflammation, hypoxemia, and impaired ventilation. Comprehensive analyses were performed, including lung histology (HE staining), bronchoalveolar lavage fluid (BALF) analysis, cytokine quantification (BCA and ELISA), pH measurement, vascular permeability (Evans Blue dye, FITC‐dextran), and blood gas analysis. Transcriptome sequencing and Western blotting revealed evidence of PANoptosis, with markers of pyroptosis, apoptosis, and necroptosis identified. Recovery from lung injury was observed at 72 h post‐induction. This model serves as a clinically relevant tool for studying aspiration pneumonia pathophysiology and therapeutic interventions.

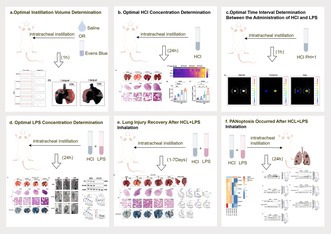

## 1 INTRODUCTION

Gastric aspiration occurs when gastric contents enter the respiratory tract via the oropharynx, a serious issue often affecting critically ill patients, such as those with impaired consciousness from trauma or stroke, and is common in ICU and emergency settings. It can be life‐threatening and insidious, with studies showing 88.9% of ICU patients experiencing at least one event during hospitalization.^1^ It also occurs in 1 in 2000–3000 general anesthesia cases.^2^, ^3^, ^4^, ^5^ Management involves immediate airway suctioning to remove aspirated material, oxygen therapy or mechanical ventilation for hypoxemia, and antibiotics if infection is suspected. Anti‐inflammatory treatments and supportive care, including fluid management and monitoring for ARDS, are critical for reducing lung injury and improving outcomes. The pathological process of aspiration pneumonia can be divided into two stages. The first stage involves damage to the pulmonary epithelial tissue caused by gastric acid, especially during Mendelson's syndrome, leading to chemical pneumonitis. The second stage is the inflammatory response triggered by pathogenic bacteria in the gastric contents. Hydrochloric acid is the main component of gastric acid, and Gram‐negative bacteria are the primary pathogens in hospital‐acquired aspiration pneumonia cases. Based on this, we attempted to use gastric acid and LPS to simulate the pathological process of hospital‐acquired aspiration pneumonia.^6^, ^7^

To control the severity of aspiration pneumonia, establishing a suitable animal model is crucial. However, current methods for modeling aspiration pneumonia remain controversial. Commonly used models include the simple hydrochloric acid inhalation model (ACID),^8^, ^9^ the sterile gastric content particle inhalation model (SNAP),^10^, ^11^ and the hydrochloric acid/gastric content particle combined inhalation model (CASP).^12^ These models primarily simulate acute chemical lung injury caused by gastric acid inhalation but neglect the inflammatory response induced by pathogens, such as Gram‐negative bacteria, present in gastric contents. None of these models address both chemical injury and pathogen‐induced inflammation. Therefore, there is a need to develop a more clinically relevant gastric aspiration pneumonia model.

In this study, we aimed to establish a rat model of gastric aspiration pneumonia using hydrochloric acid and LPS. The model's efficacy was evaluated through general observation, pathological analysis, lung function assessment, and exploration of underlying mechanisms. Key parameters were measured to comprehensively assess the pathological features of aspiration pneumonia. Body weight changes reflected systemic health, while HE staining and BALF analysis evaluated structural lung damage and inflammation. Inflammatory cytokines indicated the severity of the inflammatory response. Evans Blue and FITC‐dextran leakage assessed pulmonary vascular permeability and barrier integrity, respectively, while blood gas analysis evaluated gas exchange impairment. Western blot analysis provided molecular insights, including the detection of PANoptosis‐related proteins.

## 2 METHODS

### 2.1 Animal

Specific pathogen‐free (SPF) level Sprague Dawley rats, both male and female, weighing 140‐160 g, were procured from Beijing SPF Biotech Co., Ltd. Prior to the experiments, all rats were provided with free access to food and water and housed in an animal facility under controlled conditions: a constant temperature of approximately 25°C, humidity levels between 55% and 60%, and a 12‐h light/dark cycle. The rats were acclimated to these conditions for 3 days to prevent physiological stress responses. The animal experiments were conducted following approval from the Ethics Committee of the Experimental Animal Department of Fudan University (No.2024‐A‐038‐01).

### 2.2 Rat tracheal intubation and tracheal instillation

Rats were anesthetized with sodium pentobarbital. A rat tracheal tube with a metal guide wire (16G, outer diameter: 2 mm, metal guide wire diameter: 0.8 mm; Yuyan Scientific Instrument, Shanghai, China) was used for tracheal intubation. A 1 mL sterile syringe was connected to the tracheal tube for instillation, ensuring the liquid was administered at a uniform speed to fully enter the lungs.^13^

### 2.3 Rat pulmonary function monitoring

The EMMS R&C pulmonary function detection system is used for pulmonary function monitoring. Briefly, after successful intubation, the rat was placed under a body scanner. The tracheal cannula was connected to the interface of the body scanner, and “Rrcoad” is clicked to for data recording.^14^

### 2.4 Evans Blue distribution and leakage in lung tissue

Rats were anesthetized and given a 3% Evans Blue solution via tracheal instillation. Lung tissue was collected 1 h later to check for blue coloration. For leakage analysis, rats were administered saline, different concentrations of HCl, or LPS via tracheal instillation (*n* = 6). After 24–72 h, the rats were euthanized, and a 3% Evans Blue solution was injected via the tail vein. The lungs were then perfused with saline, extracted, incubated in formamide at 60°C for 24 h, and the supernatant was analyzed for Evans Blue concentration at a wavelength of 630 nm.^15^

### 2.5 Analysis of BALF protein concentration and inflammatory cytokines

Rats were administered saline, different concentrations of HCl, or LPS via tracheal instillation (*n* = 6) and euthanized 24–72 h later. The neck skin was cut to expose the trachea. Using a 5 mL syringe with a needle, 5 mL of sterile saline was injected into the trachea to evenly inflate the lungs, which were then left to rest for 30 s before the liquid was slowly withdrawn. This process was repeated three times, and the supernatant was collected after centrifugation. The protein concentration in the bronchoalveolar lavage fluid (BALF) was measured using the BCA method, and inflammatory cytokine levels in the BALF were determined using commercial ELISA kits (ImmunoWay Biotechnology, Shanghai, China), following the manufacturer's instructions.^16^, ^17^

### 2.6 Lung tissue sampling, HE staining and lung injury scoring

Rats were administered saline, different concentrations of HCl, or LPS via tracheal instillation (*n* = 6) and euthanized 24–72 h later. The chest skin was quickly dissected to expose the lung tissue. The lungs were carefully excised, fixed in 4% paraformaldehyde for 24 h, and embedded in paraffin. Thin sections (4–6 μm) were prepared, stained with hematoxylin and eosin (HE), dehydrated, cleared, and coverslipped. The stained sections were examined under an optical inverted microscope, and 20 random high‐power fields were selected in a blinded manner for scoring. Lung injury was assessed based on alveolar wall thickening, inflammatory cell infiltration, hemorrhage, and exudate presence. Scoring was performed independently by two blinded observers to ensure consistency.^18^ The lung injury scoring method is provided in Supporting Information—Table S1.

### 2.7 PH determination of BALF

The above collected BALF was mixed with Methyl Red‐Bromocresol Green indicator, and placed on a white background plate for colorimetric comparison with the standard.^19^

### 2.8 FITC‐dextran leakage experiment

Rats inhaled saline or hydrochloric acid (pH = 1,1.5 μL/g.wt) and were immediately injected with FITC‐dextran (100 μg/g.wt) via the tail vein(*n* = 3). One hour after the intervention, the lung tissue was quickly removed and placed in an animal live imaging system (Yuyan Scientific Instrument, Shanghai, China) for photography and data recording.^20^

### 2.9 Survival analysis

After intervention, the control rats and model rats were placed in cages in a constant temperature and humidity environment. The number of dead and remaining rats was recorded according to the experimental requirements (*n* = 10).^21^

### 2.10 Blood gas analysis of rats

After euthanizing the rats, blood was quickly collected from the abdominal aorta and placed in an arterial blood gas analyzer (RADIOMETER) for analysis (*n* = 6).^22^

### 2.11 Electron microscopy observation

Rats were euthanized 24 h after inhaling saline or HCl (pH = 1,1.5 μL/g.wt) + LPS (20 μg/g.wt). Samples were quickly collected, fixed, and dehydrated. The samples were then prepared as ultrathin sections and mounted on copper grids. These sections were observed under a transmission electron microscope (BRESSER, Germany) with uranium staining, and photographs were taken.^23^

### 2.12 Western blot

Rats were euthanized 24 h after inhaling saline or HCl (pH = 1) + LPS (20 μg/g.wt) (*n* = 3), and lung tissue was collected and immediately ground in liquid nitrogen. The supernatant was collected after centrifugation and subjected to protein denaturation, SDS‐PAGE gel electrophoresis, membrane transfer, blocking with milk powder, and incubation with primary and secondary antibodies. Band exposure was performed using EZL solution, and grayscale quantification was conducted using ImageJ software.^24^ All reagents for Western blot experiments were obtained from Shanghai Epizyme Biotech, and the antibodies used are listed in Supporting Information—Table S2.

### 2.13 Transcriptome detection and bioinformatics analysis

Rats were euthanized 24 h after inhaling saline or HCl (pH = 1) + LPS (20 μg/g body weight) (*n* = 3), and lung tissues were collected and rapidly frozen in liquid nitrogen. Transcriptome sequencing was then performed by Shanghai Ribiology Biotech Co., Ltd.^25^

### 2.14 Statistical analysis

All data were statistically analyzed using GraphPad Prism 8.0. Measurement data was expressed as mean ± standard deviation. Multiple group comparison was process using one‐way ANOVA analysis, followed by post‐hoc comparison using a LSD test. Kaplan–Meier curves were constructed for survival analysis. *p<0.05, **p<0.01, ***p<0.001, ****p<0.0001 were considered statistically significant.

## 3 RESULTS

### 3.1 Optimal instillation volume determination

**FIGURE 1:**
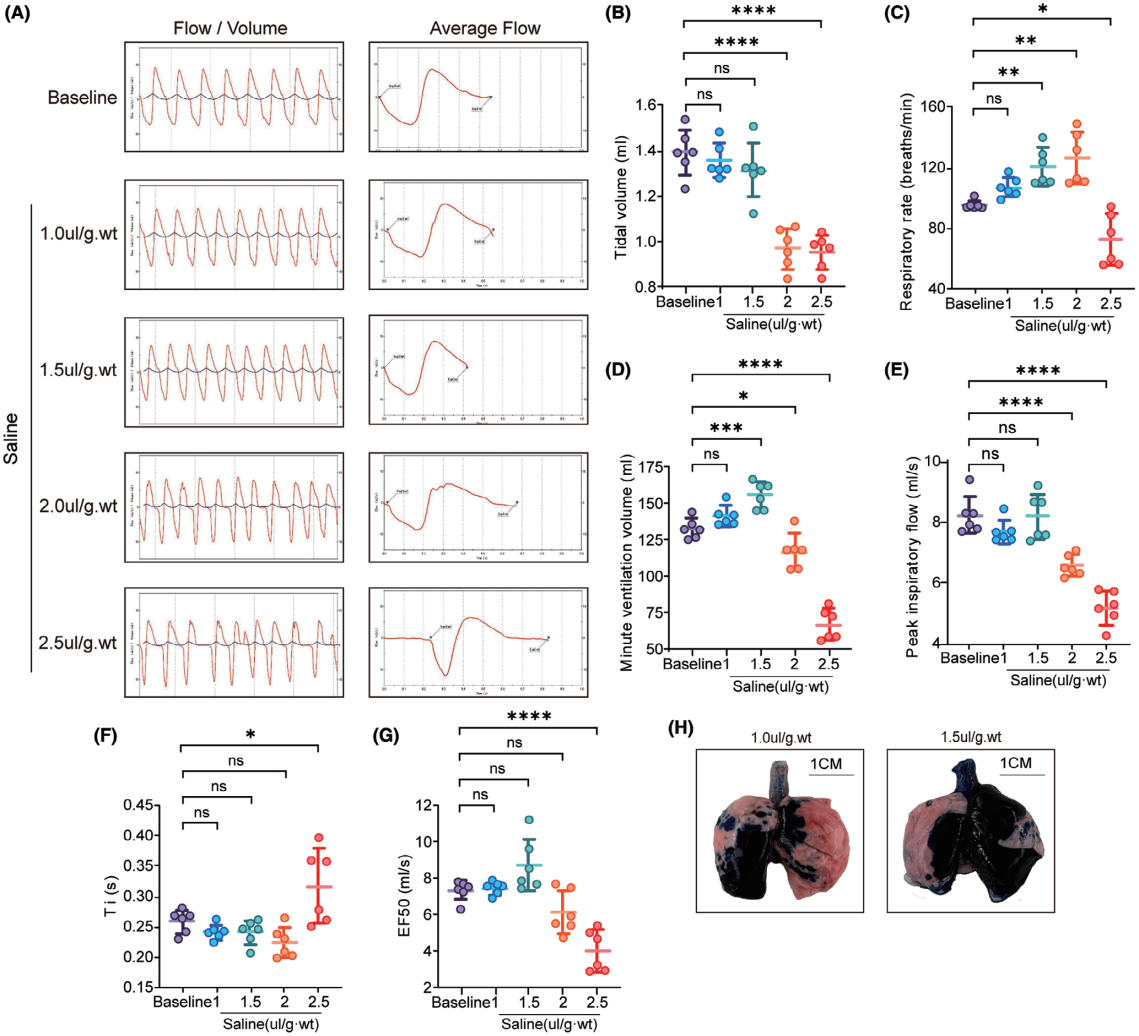
Optimal infusion volume determination. (A) Representative figures of respiratory flow rate curve (red) and tidal volume curve (blue) of the rat at baseline and after instillation of different volumes of saline. (B–G) Quantification of the lung function indexes at baseline and after instillation of different volumes of saline, *n* = 6: (B) tidal volume; (C) respiration rate; (D) minute ventilation; (E) peak inspiratory flow rate; (F) inspiratory time; (G) inspiratory flow rate at 50% maximal inspiratory volume (EF50); (H) Lung appearance after inhaling of different volumes of Evans Blue solutions. Data are presented as the mean SEM, with each point representing an independent experiment. *p < 0.05, **p < 0.01, ***p < 0.001, ****p < 0.0001 versus the matched group.

To investigate the optimal volume for intratracheal instillation, we tested four different volumes of sterile saline: 1 μL/g.wt, 1.5 μL/g.wt, 2 μL/g.wt, and 2.5 μL/g.wt, and monitored changes in lung function post‐instillation in rats. At 1 μL/g.wt and 1.5 μL/g.wt, an increased respiratory rate with a normal rhythm was observed. However, at 2 μL/g.wt and 2.5 μL/g.wt, changes in respiratory rhythm were detected, including noticeable apnea due to upper airway obstruction, particularly at 2.5 μL/g.wt (Figure 1A). Additional lung function parameters related to airway obstruction were also assessed. Compared to baseline, no significant changes in tidal volume, peak inspiratory flow, inspiratory time, and EF50 were observed at 1 μL/g.wt and 1.5 μL/g.wt. At 2.0 μL/g.wt, despite a significant increase in respiratory rate, tidal volume and minute ventilation decreased. At 2.5 μL/g.wt, tidal volume, respiratory rate, minute ventilation, peak inspiratory flow, and EF50 significantly decreased, while inspiratory time notably increased. These findings indicate that instillation volumes of 1 μL/g.wt and 1.5 μL/g.wt had minimal impact on respiratory function (Figure 1B–G). To verify whether 1 μL/g.wt or 1.5 μL/g.wt could reach deep lung tissue, Evans Blue dye was used. One hour post‐instillation, a more uniform and extensive distribution was observed at 1.5 μL/g.wt (Figure 1H). In summary, an instillation volume of 1.5 μL/g.wt was identified as optimal, achieving uniform lung distribution with minimal impact on respiratory function.

### 3.2 Optimal HCl concentration determination

**FIGURE 2:**
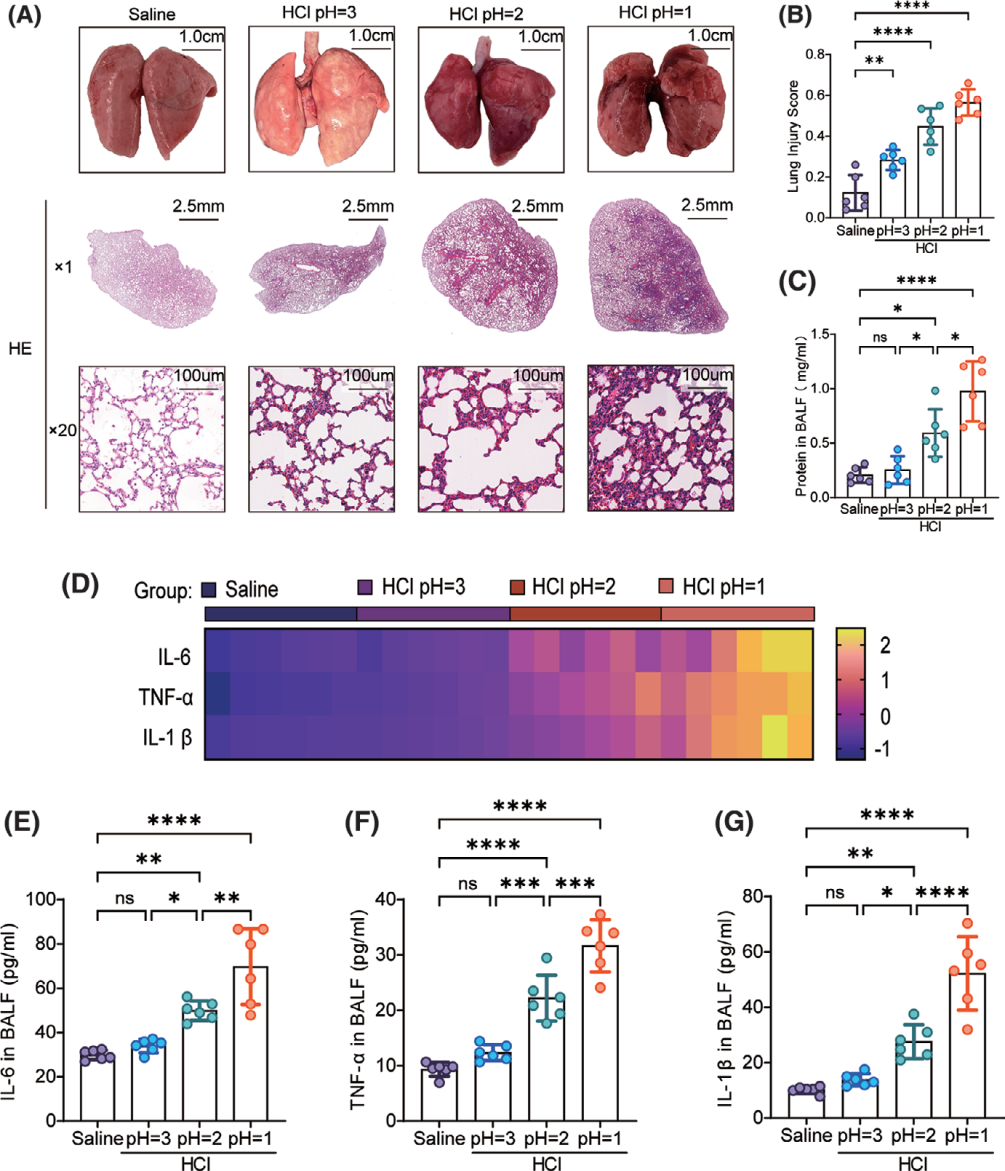
Optimal HCl concentration determination. (A) Lung appearance and HE staining after inhalation of normal saline and different concentrations of hydrochloric acid. (B) Lung injury score, *n* = 6. (C) BALF protein concentration, *n* = 6. (D) Heatmap of inflammatory factor concentration. (E–G) BALF inflammatory factors levels, *n* = 6. Data are presented as the mean SEM, with each point representing an independent experiment. *p < 0.05, **p < 0.01, ***p < 0.001, ****p < 0.0001 versus the matched group.

To determine the optimal concentration of hydrochloric acid, the main component of stomach acid, we studied its effects on lung tissue damage caused by gastric juice inhalation. Hydrochloric acid at three different concentrations (pH = 1–3) was infused into the rat trachea. After 24 h, lung tissue was evaluated for appearance and pathology. Compared to control rats, those treated with pH = 1 hydrochloric acid exhibited obvious pulmonary edema near the lung hilum and significant changes in lung color, whereas no apparent injury was observed in the pH = 3 group. Additionally, HE staining of lung tissue showed thickened alveolar walls, significant eosinophil infiltration, and protein fragments in the alveolar spaces in the pH = 1 group. The lung injury score confirmed the highest pathological scores in the pH = 1 hydrochloric acid group (Figure 2 A, B). Inhalation of hydrochloric acid also led to pulmonary capillary dysfunction, with significantly increased protein levels in the BALF in the group treated with pH = 1 (Figure 2C). Moreover, hydrochloric acid inhalation caused a concentration‐dependent increase in inflammatory factors in the BALF (Figure 2D). When the pH = 1, levels of IL‐6, IL‐1β, and TNF‐α in the BALF were significantly elevated (Figure 2E–G). The results indicate that hydrochloric acid at pH = 1 was the most effective concentration for inducing significant lung injury without causing excessive harm.

### 3.3 Optimal time interval determination between the administration of hydrochloric acid and LPS

**FIGURE 3:**
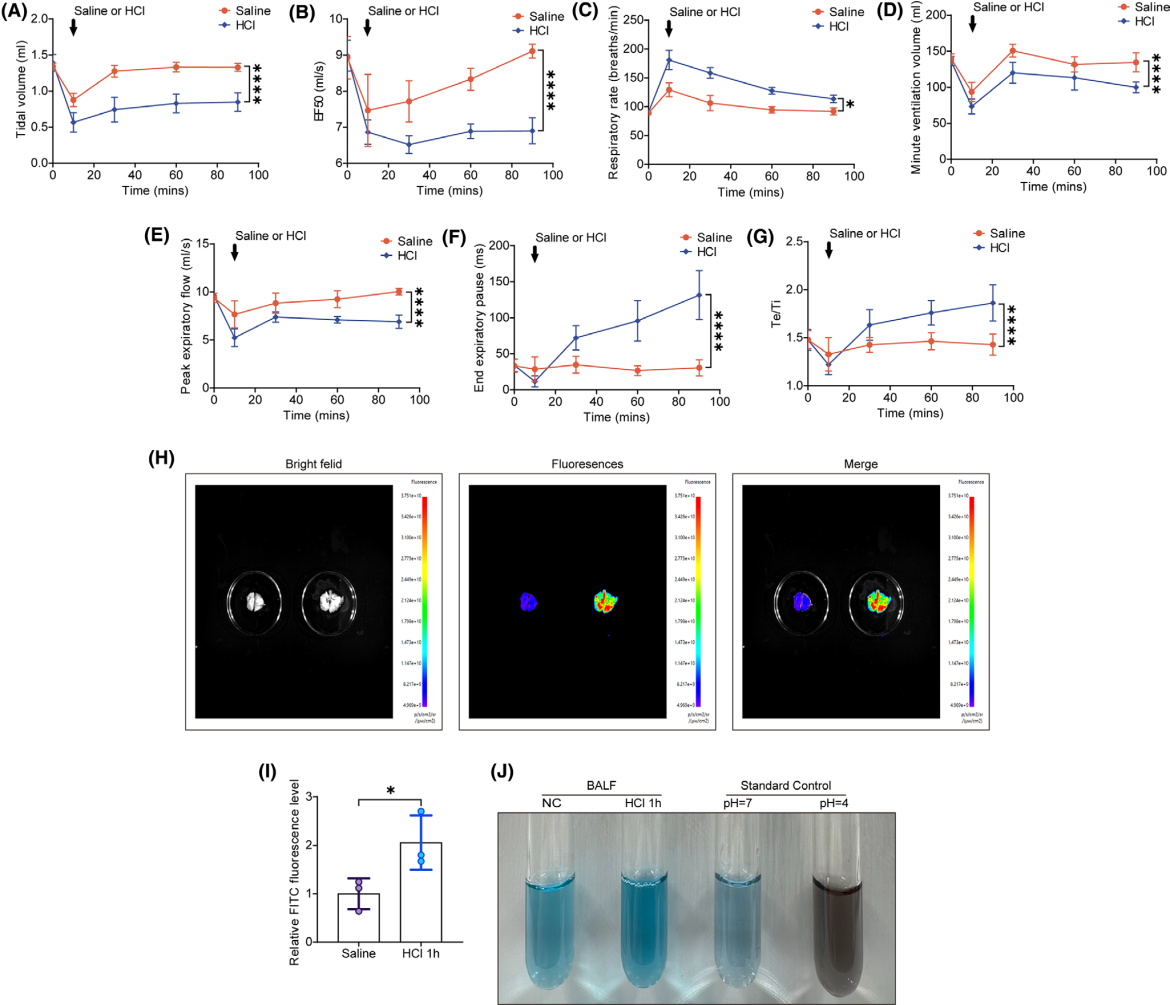
Lung function assessment after saline and hydrochloric acid instillation. (A–G) Continuous lung function monitoring in rats 90 min after tracheal instillation of hydrochloric acid (pH = 1), *n* = 6: (A) tidal volume; (B) inspiratory flow rate at 50% maximal inspiratory volume (EF50); (C) respiration rate; (D) minute ventilation; (E) peak inspiratory flow rate; (F) end expiratory pause; (G) the ratio of expiratory time to inspiratory time; lung and vascular permeability determined by FITC‐Dextran leakage. (H) Representative images. Colors represent relative fluorescence intensity, with blue indicating the lowest and red indicating the highest. (I) Fluorescence quantification, *n* = 3. (J) BALF pH determination. Data are presented as the mean SEM, with each point representing an independent experiment. *p < 0.05, ****p < 0.0001 versus the matched group.

The secondary damage caused by the inflammatory response from inhaled pathogens was simulated through intratracheal instillation of LPS. Mixing LPS with hydrochloric acid at pH = 1 can lead to LPS hydrolysis. Clinically, the delayed inflammatory response induced by inhaled pathogens often follows chemical lung damage caused by gastric acid. To determine the optimal timing for LPS administration, we continuously monitored lung function in rats after hydrochloric acid inhalation. Compared to the saline group, significant reductions in tidal volume, minute ventilation, peak expiratory flow rate, end‐expiratory pause, and EF50 were observed, along with increased expiratory/inspiratory time ratio and respiratory frequency (Figure 3A–G). These changes indicate that hydrochloric acid inhalation leads to respiratory distress, airway hyper‐responsiveness, and impaired pulmonary ventilation. These changes gradually stabilized approximately 1 h after hydrochloric acid inhalation (although they did not return to normal), suggesting that administering LPS at this point can avoid excessive mortality and effectively simulate the inflammatory response secondary to hydrochloric acid‐induced chemical lung injury. Therefore, we concluded that 1 h after hydrochloric acid inhalation is the optimal time for LPS administration. To confirm significant chemical lung damage during LPS infusion, we measured FITC dextran leakage in lung tissue 1 h post‐hydrochloric acid instillation. A significant increase in FITC dextran leakage indicated severe damage to the pulmonary microvascular barrier and substantial chemical lung injury (Figure 3H,I). Additionally, to ensure LPS was not hydrolyzed by hydrochloric acid, we monitored the pH of the BALF 1 h post‐instillation. Colorimetric results showed no significant change in lung pH, remaining similar to baseline levels (Figure 3J). The findings show that a 1 h interval between HCl and LPS administration ensured stable lung function and elicited the strongest inflammatory response.

### 3.4 Optimal LPS concentration determination

**FIGURE 4:**
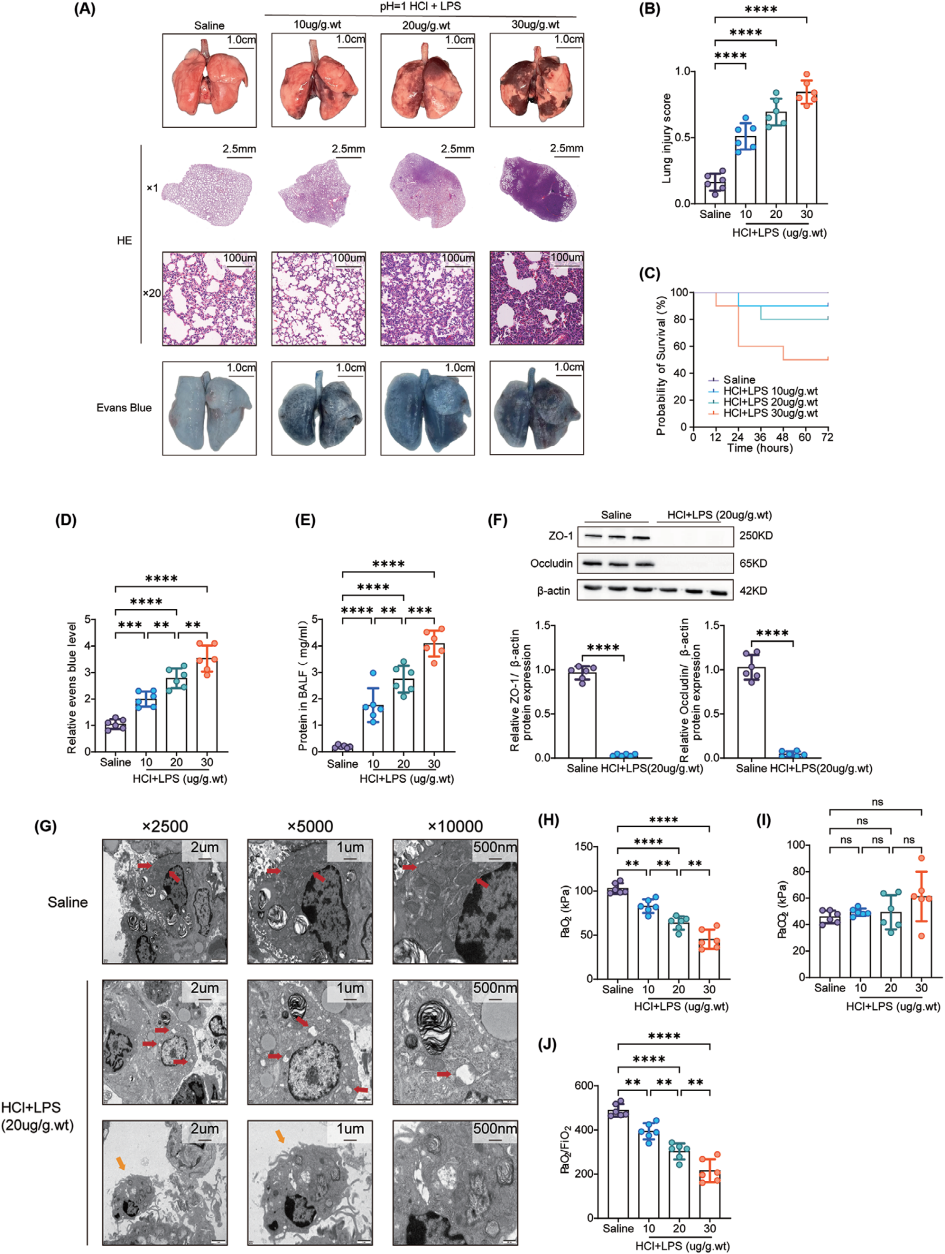
Optimization of LPS concentration for model establishing. (A) Lung appearance and HE staining pathology; Evans Blue evaluation after inhalation of normal saline and hydrochloric acid plus different concentrations of LPS. (B) Lung injury score, *n* = 6; (C) Survival curve, *n* = 10. (D) Evans Blue levels, *n* = 6. (E) BALF protein level, *n* = 6. (F) Western‐blotting determination of the cell junction proteins ZO‐1 and Occludin, *n* = 6; (G) Ultra micro‐structure of the lung determined by electronic microscopy. Red arrows: Type II alveolar epithelial mitochondria. Yellow arrows: Macrophages free in the alveolar space. (H) The partial pressure of oxygen (PaO2). (I) The partial pressure of carbon dioxide (PaCO2). (J) PaO2/fraction of oxygen in inspired air (FIO2), *n* = 6. Data are presented as the mean SEM, with each point representing an independent experiment. *p < 0.05, **p < 0.01, ***p < 0.001, ****p < 0.0001 versus the matched group.

**FIGURE 5:**
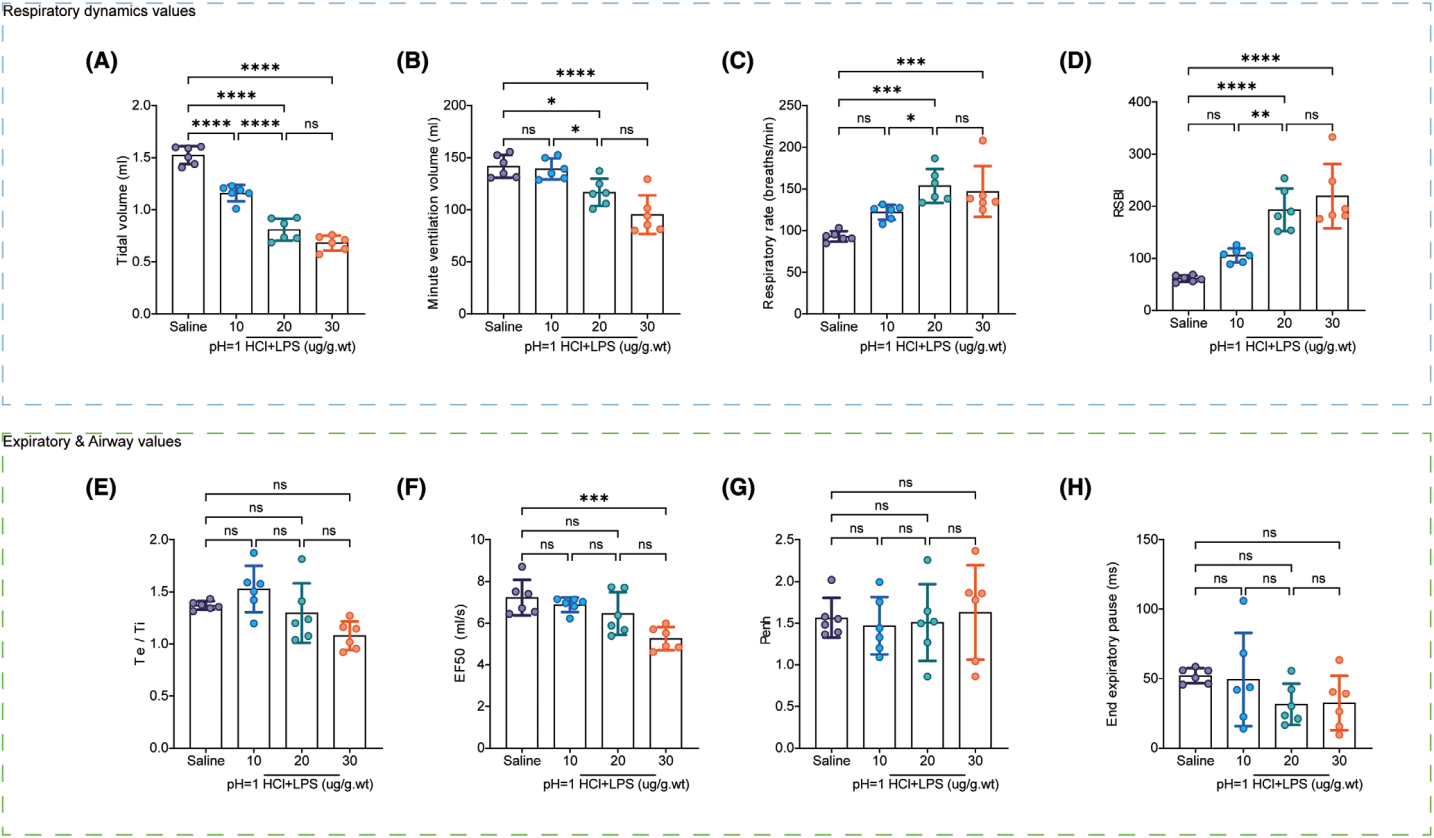
Effects of hydrochloric acid plus LPS on the lung function. (A) Tidal volume. (B) Minute ventilation. (C) Respiration rate. (D) The rapid shallow breathing index (RSBI). (E) The ratio of expiratory time to inspiratory time. (F) Inspiratory flow rate at 50% maximal inspiratory volume (EF50). (G) Enhanced pause (Penh). (H) End expiratory pause peak inspiratory flow rate, *n* = 6. Data are presented as the mean SEM, with each point representing an independent experiment. *p < 0.05, **p < 0.01, ***p < 0.001, ****p < 0.0001 versus the matched group.

To determine the optimal LPS concentration for inducing lung injury, we tested doses of 10 μg/g.wt, 20 μg/g.wt, and 30 μg/g.wt following a 1‐h pre‐treatment with 1.5 μL/g.wt hydrochloric acid at pH = 1. All treated groups exhibited significant lung damage, such as edema, hemorrhage, and color changes, which intensified with increasing LPS doses (Figure 4A,B). HE staining confirmed these findings, showing eosinophilic cell infiltration and alveolar wall thickening. The 20 μg/g.wt LPS group displayed characteristics of ARDS, such as intra‐alveolar hemorrhage, while the 30 μg/g.wt LPS group experienced severe lung damage, including extensive alveolar destruction and edema. Lung injury scores corroborated these observations (Figure 4A,D), and the Evans Blue dye assay indicated a dose‐dependent increase in lung tissue leakage (Figure 4A,E). We also assessed 72‐h survival rates for each group, finding that the HCl + LPS (30 μg/g.wt) group had only a 50% survival rate. This high mortality suggests that the dose was excessively severe, whereas the 20 μg/g.wt LPS dose caused significant lung damage with a higher survival rate, making it suitable for modeling (Figure 4C). Transmission electron microscopy (TEM) revealed swollen mitochondria and disrupted cristae in alveolar epithelial cells, along with alveolar macrophages in the HCl + 20 μg/g.wt LPS group (Figure 4G). The reduction in tight junction proteins ZO‐1 and Occludin (Figure 4F), and the increased protein levels in BALF indicated barrier dysfunction (Figure 4E). Arterial blood gas analysis showed reduced oxygenation capacity in all LPS groups, with no significant differences in carbon dioxide levels (Figure 4H–J). Lung function tests indicated decreased tidal volume, reduced minute ventilation, and increased respiratory rate. The absence of significant changes in small airway function suggests that the respiratory distress in the acute phase of this model primarily reflects impaired alveolar gas exchange, similar to clinical ARDS (Figure 5A–H). A dose of 20 μg/g.wt LPS provided the best balance between significant lung injury and a higher survival rate, making it the most suitable concentration for the model.

### 3.5 Lung injury recovery after HCL + LPS inhalation

**FIGURE 6:**
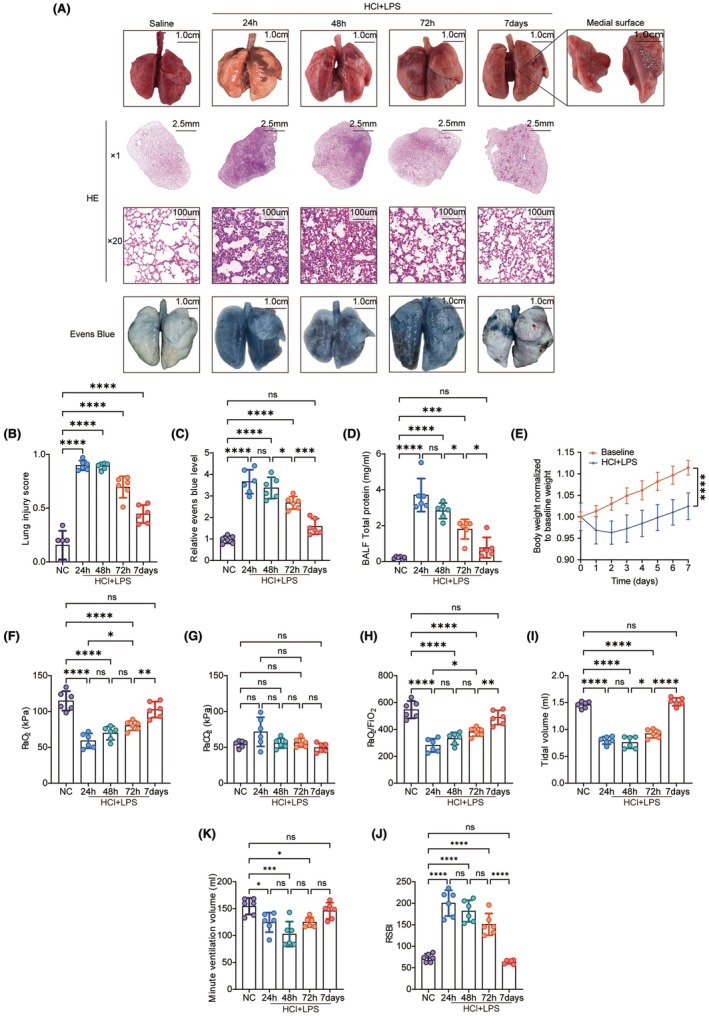
Time course (24 h—7 days) of the lung appearance, pathology and function after hydrochloric acid plus LPS installation. (A) Time course of lung appearance and HE staining pathology; Evans Blue evaluation after inhalation of normal saline and hydrochloric acid plus LPS; (B) Lung injury score, *n* = 6. (C) Relative levels of Evans Blue, *n* = 6. (D) BALF protein levels, *n* = 6. (E) Body weight monitoring, *n* = 6. (F) The partial pressure of oxygen (PaO2), *n* = 6; (G) The partial pressure of carbon dioxide (PaCO2), *n* = 6. (H) PaO2/FiO2, *n* = 6. (I) Tidal volume, *n* = 6. (J) The rapid shallow breathing index (RSBI), *n* = 6. (K) minute ventilation, *n* = 6. Data are presented as the mean SEM, with each point representing an independent experiment. *p < 0.05, **p < 0.01, ***p < 0.001, ****p < 0.0001 versus the matched group.

In the study mentioned above, we successfully established a rat model of aspiration pneumonia and confirmed acute lung injury at 24 h. To evaluate the model's effectiveness in studying lung injury recovery, we observed the lung injury at 24 h, 48 h, 72 h, and 7 days post‐exposure. At 24 h post‐exposure, the lung tissue showed significant changes compared to baseline, including discoloration, edema, and hemorrhage. The damage observed at 48 h was similar to that at 24 h. However, by 72 h, there was a marked reduction in edema, hemorrhage, and discoloration. By Day 7, significant improvement was observed, although partial and incomplete recovery near the hilum persisted (Figure 6A). These findings were confirmed by histological examination using HE staining, which revealed extensive infiltration of eosinophilic inflammatory cells, thickened alveolar walls, protein exudation, and alveolar hemorrhage at 24 and 48 h. By 72 h, these symptoms had alleviated. By Day 7, the lung tissue showed significant recovery, with reduced eosinophilic inflammatory cell infiltration and near‐restoration of the alveolar structure to baseline levels, as indicated by lung injury scores (Figure 6A,B). Furthermore, the Evans Blue leakage assay demonstrated a decrease in lung tissue leakage beginning at 72 h (Figure 6C). Similarly, protein content in bronchoalveolar lavage fluid (BALF) significantly increased at 24–48 h but began to normalize by 72 h, showing no significant difference compared to baseline by Day 7 (Figure 6D). Body weight changes in rats also reflected lung damage and recovery. Compared to the control group, rats experienced significant weight loss within 24–48 h after HCl + LPS inhalation. However, this trend reversed by 72 h, with weight gain observed from Day 3 to day 7, indicating the onset of recovery (Figure 6E). Recovery from hypoxemia and pulmonary ventilation dysfunction, common clinical indicators of aspiration pneumonia, was monitored using arterial blood gas analysis. At 24 h post‐exposure, arterial oxygen partial pressure and oxygenation index were significantly reduced. These values gradually improved over the following days, returning to baseline levels by Day 7 (Figure 6F–H). Lung function tests showed decreased tidal volume, increased respiratory rate, and a higher shallow rapid breathing index at 24–48 h post‐exposure. However, these parameters began to normalize by 72 h, showing no significant difference from baseline by Day 7 (Figure 6I–K). The observations suggest that the recovery phase became most apparent at 72 h post‐injury, marking this as a critical time point for evaluating lung repair.

### 3.6 PANoptosis occurred after HCL + LPS inhalation

**FIGURE 7:**
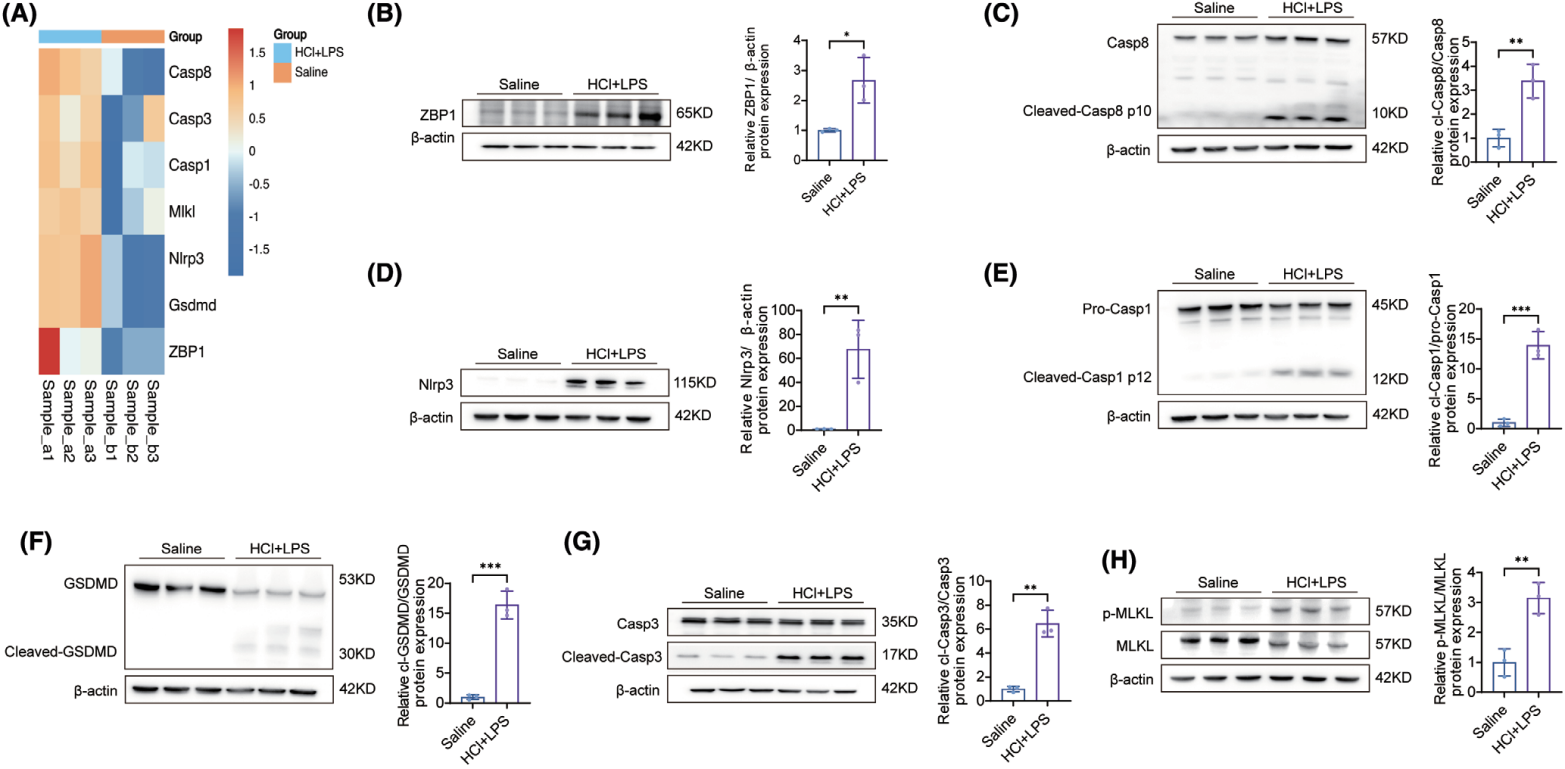
Panoptosis processes, including evaluation of apoptosis, pyroptosis and necroptosis proteins. (A) RNA‐seq Heatmap of the cell programmed death in saline control and LPS + HCl model group, *n* = 3. (B–H) Representative Western blotting images of PANoptosis Markers and β‐Actin, and quantitative analysis were performed using ImageJ software, *n* = 3: (B) ZBP1 protein; (C) cleaved Caspase‐8; (D) NLRP3; (E) cleaved Caspase‐1; (F) cleaved GSDMD; (G) cleaved Caspase‐3; (H) MLKL phosphorylation. Data are presented as the mean SEM, with each point representing an independent experiment, *p < 0.05, **p < 0.01, ***p < 0.001 versus the matched group.

PANoptosis is a newly identified programmed cell death mechanism associated with various pathological conditions, including acute respiratory distress syndrome (ARDS) and lung injury. LPS can induce PANoptosis, leading to tissue damage, particularly in cases such as sepsis. To investigate whether PANoptosis also occurs in gastric aspiration pneumonia, we employed the modeling method mentioned earlier. Lung tissue was collected 24 h after rats were exposed to HCl (pH = 1) + LPS (20 μg/g.wt) for analysis. RNA‐seq analysis revealed a significant increase in PANoptosis‐related RNA molecules compared to the control group (which inhaled saline) (Figure 7A). Western blot analysis further confirmed this result, showing elevated levels of PANoptosis markers such as ZBP1, Caspase8, Cleaved‐Caspase8, Pro‐Caspase1, and NLRP3 proteins in rats exposed to HCl + LPS (Figure 7B–E). Additionally, proteins associated with apoptosis, necrosis, and pyroptosis, including Caspase3, Cleaved‐Caspase3, GSDMD, Cleaved‐GSDMD, MLKL, and p‐MLKL, were significantly increased (Figure 7F–H). These results confirm that PANoptosis occurred in the lung tissue of rats after HCl + LPS exposure. Overall, the combined treatment of HCl (PH = 1)and 20 μg/g.wt LPS effectively induced PANoptosis, highlighting the molecular relevance of this model for studying the mechanisms of programmed cell death in lung injury.

## 4 DISCUSSION

Based on the parameters measured, the proposed mechanism of lung injury in this aspiration pneumonia model involves a two‐phase process. Initially, intratracheal instillation of HCl induces acute chemical injury to the lung epithelium, evident from histological changes (HE staining), increased protein concentration in BALF, and elevated vascular permeability (Evans Blue and FITC‐dextran leakage). This damage disrupts the alveolar‐capillary barrier, as reflected by blood gas analysis showing impaired gas exchange and hypoxemia.

The second phase is characterized by an inflammatory response triggered by lipopolysaccharide (LPS), representing pathogen‐induced inflammation. This phase involves elevated levels of inflammatory cytokines in BALF, further exacerbating lung injury through the recruitment of immune cells and increased pulmonary microvascular permeability. Western blot analysis indicates the activation of PANoptosis, a programmed cell death pathway involving apoptosis, necrosis, and pyroptosis, which amplifies tissue damage and inflammation.

These two phases—chemical injury followed by pathogen‐induced inflammation—act synergistically to replicate the clinical progression of aspiration pneumonia. The model highlights the critical role of microvascular dysfunction and inflammatory signaling in lung injury. Understanding this mechanism provides valuable insights for targeting specific pathways, such as mitigating PANoptosis or strengthening the alveolar‐capillary barrier, in the development of therapeutic strategies.^26^

To simulate the clinical aspiration process, this study utilized a method of tracheal instillation. Rats inhaled hydrochloric acid or LPS through spontaneous breathing, allowing these substances to reach the alveoli via the trachea, causing tissue damage. Due to the influence of lung secretions, the concentration of these substances diluted over time, resulting in varying degrees of damage across different lung regions. In this study, we first optimized the volume of liquid for tracheal instillation. It was found that volumes exceeding 2 μL/g.wt led to upper airway obstruction and suffocation. Conversely, volumes less than 1 μL/g.wt did not distribute evenly in the deep lung alveolar tissue, reducing the extent of lung damage or confining it to areas near the hilum. Therefore, a volume of 1.5 μL/g.wt was determined to be optimal for achieving uniform distribution within the lungs and alveoli. This volume also serves as a reference for other rat models requiring tracheal instillation.

Previous studies have shown that intratracheal instillation of hydrochloric acid can cause acute lung injury, with chemical pneumonia typically resolving within 24 h.^27^, ^28^, ^29^ Our data indicate that the severity of chemical lung injury is concentration dependent. Inhalation of gastric acid with a pH greater than 2.5 did not cause significant acute chemical pneumonia, while our physiological pH for gastric acid is greater than 0.9. This study found that inhalation of hydrochloric acid with a pH of 1 induced noticeable chemical pneumonia features at 24 h, including alveolar tissue damage, pulmonary microvascular dysfunction, and a significant inflammatory response. Thus, this study selected hydrochloric acid with pH = 1 to model gastric acid inhalation and induce significant chemical lung injury. Furthermore, this study observed significant pulmonary microvascular dysfunction as early as 1 h post‐inhalation of hydrochloric acid. Airway hyperresponsiveness and ventilation‐related issues were noted, stabilizing around 60 min post‐inhalation, making this the optimal time for LPS administration. The chosen 60‐min interval ensures lung function stability, optimizing experimental outcomes.

Similar to hydrochloric acid, LPS‐induced lung injury was also concentration dependent. However, high concentrations of LPS (30 μg/g.wt) led to an excessively high mortality rate within 72 h. Therefore, this study determined that a concentration of 20 μg/g.wt LPS was optimal. This dose resulted in severe lung injury within 24 h post‐inhalation, characterized by significant alveolar damage and an inflammatory response, similar to clinical ARDS. The injury also included pulmonary microvascular dysfunction, significant hypoxemia, and respiratory distress. Additionally, these rats primarily exhibited symptoms consistent with type I respiratory failure, where the primary issue is pulmonary ventilation dysfunction. This finding provides new insights for research and clinical treatment of gastric content aspiration pneumonia.

**FIGURE 8:**
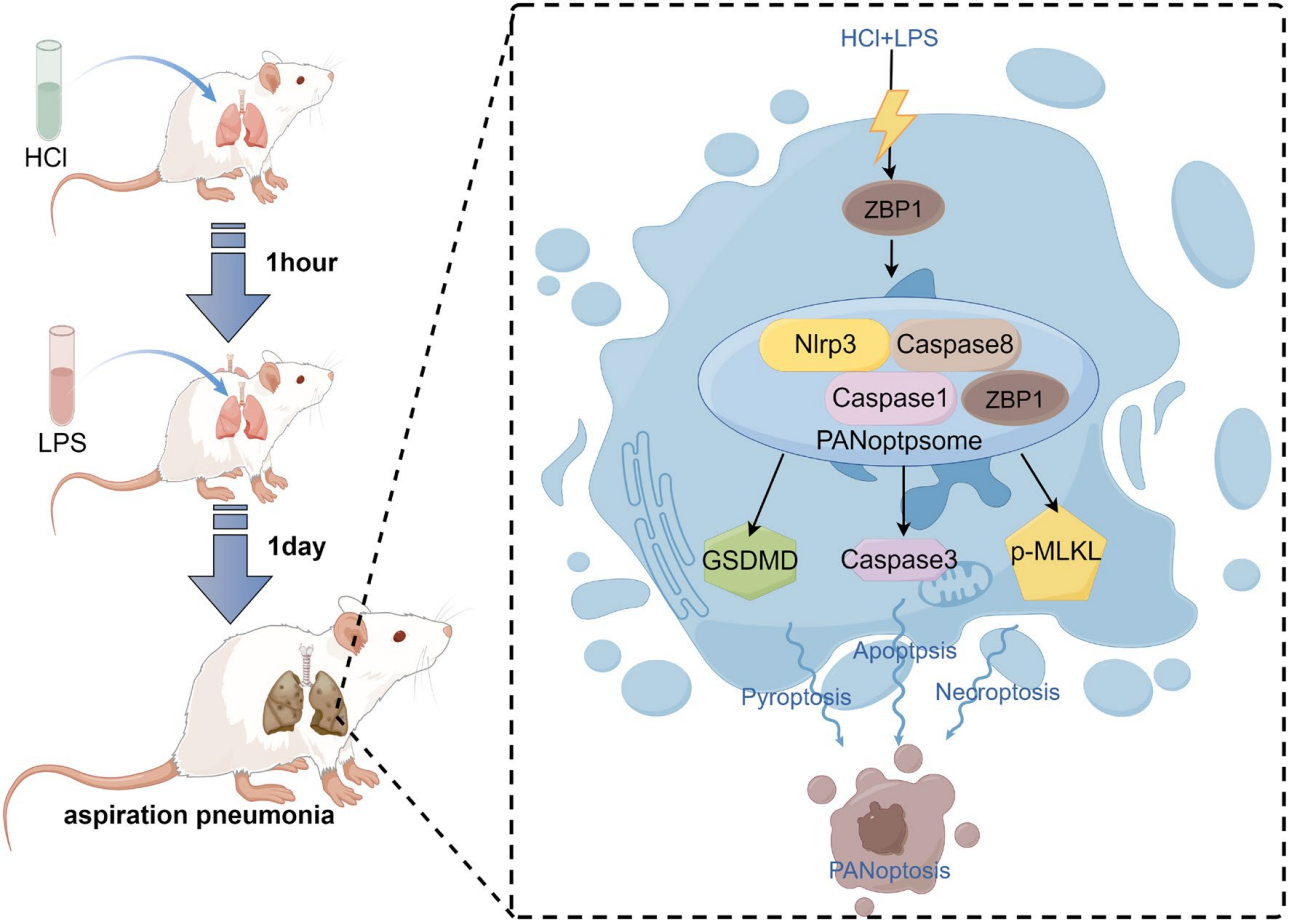
Schematic diagram. In this study, a rat model of aspiration pneumonia was established using hydrochloric acid (pH = 1, 1.5 μL/g body weight) and LPS (20 μg/g body weight), and PANoptosis was detected in the lung tissue of rats with aspiration pneumonia.

Additionally, this study conducted a 7‐day observation study and found that the model remained in the acute phase of lung injury for 24–48 h, with the most severe damage occurring during this period. From 72 h onward, the lung injury gradually entered the recovery phase.^30^ These findings confirm that this model can be used to study the acute phase of gastric content aspiration pneumonia and the tissue repair mechanisms during recovery. We also observed the occurrence of PANoptosis in this model, a programmed cell death mechanism. PANoptosis is recognized as a critical factor in acute lung injury.^31^ To further elucidate the molecular underpinnings of PANoptosis and its role in lung injury, this study investigated the involvement of key proteins, including Caspase8, Caspase1, NLRP3, Caspase3, GSDMD, and MLKL. Caspase8 serves as a critical initiator of PANoptosis, integrating apoptosis, pyroptosis, and necroptosis pathways while activating downstream inflammatory responses.^32^ NLRP3 forms the inflammasome in response to cellular damage, leading to the activation of Caspase1, which processes pro‐inflammatory cytokines IL‐1β and IL‐18 and induces pyroptosis.^33^ GSDMD, cleaved by Caspase1 or Caspase8, forms membrane pores, resulting in cell lysis and the release of inflammatory mediators that amplify lung damage. Caspase3, as the executor of apoptosis, mediates epithelial cell death, disrupting lung structure and function. MLKL, the terminal effector of necroptosis, disrupts cell membranes and releases damage‐associated molecular patterns (DAMPs), further intensifying inflammatory responses. These findings underscore the complex interplay among apoptosis, pyroptosis, and necroptosis in gastric aspiration pneumonia. The interactions of these molecular pathways drive inflammation, exacerbate tissue damage, and impair lung function, providing potential targets for therapeutic intervention in this condition^34^, ^35^ (Figure 8). This study has some limitations, notably that the rats used were all young, aged 4–6 weeks. This age group may respond differently to injury and repair processes compared to older rats, potentially limiting the model's applicability to older populations.

## 5 CONCLUSION

The optimal conditions for modeling gastric aspiration pneumonia in rats were identified as 1.5 μL/g.wt of hydrochloric acid at a pH of 1, followed by 20 μg/g.wt of lipopolysaccharide (LPS) administered 1 h later. This combination effectively replicates the two main pathological features of gastric aspiration pneumonia: chemical lung injury caused by gastric acid and an inflammatory response induced by pathogens. The selected dosages induced significant lung damage and inflammation while maintaining an acceptable survival rate, providing a robust and clinically relevant model for studying the pathophysiology and therapeutic strategies of this condition. In conclusion, the optimized model, involving 1.5 μL/g.wt of hydrochloric acid (pH = 1) followed by 20 μg/g.wt of LPS after 1 h, successfully replicated key pathological features of aspiration pneumonia, including acute lung injury and inflammatory responses.

## AUTHOR CONTRIBUTIONS

**Hanbing Hu:** Methodology; writing – original draft; writing – review and editing. **Junfeng Chen:** Investigation; methodology. **Yiru Shao:** Writing – review and editing. **Yuedong Tang:** Writing – review and editing. **Yu Dun:** Conceptualization; software. **Obulkasim Memet:** Supervision. **Xuanrong Bao:** Software. **Jie Shen:** Conceptualization; project administration; resources.

## FUNDING INFORMATION

The study was supported by the National Natural Science Foundation of China (No. 82272243) and the Science and Technology Commission of Shanghai Municipality (No. 22Y11900800).

## CONFLICT OF INTEREST STATEMENT

The authors declare no conflicts of interest.

## ETHICAL STATEMENT

All animal experiments were conducted in accordance with the principles of good laboratory animal care and performed in compliance with the Animal Ethics Review Committee of Fudan University(No.2024‐A‐038‐01).

## Supporting information


Table S1.
