# Supplementary material for: Establishment of rat model for aspiration pneumonia and potential mechanisms
Source: Animal Model Exp Med. 2025 Mar 20;8(6):1105–18. doi: 10.1002/ame2.12566 (PMC12205006; doi:10.1002/ame2.12566)
Supplement: Supplementary file 1 — Table S1. [file AME2-8-1105-s001.docx]

Table S1

| Parameter | Score per field | | |
| --- | --- | --- | --- |
|  | 0 | 1 | 2 |
| Neutrophils in the alveolar space | none | 1-5 | ＞5 |
| Neutrophils in the interstitial space | none | 1-5 | ＞5 |
| Hyaline membranes | none | 1 | ＞1 |
| Proteinaceous debris filling the airspaces | none | 1 | ＞1 |
| Alveolar septal thickening | ＜2x | 2x-4x | ＞4x |

Score=[(20×A)+ (14×B) + (7×C) +(7×D) +(2×E)]/(number of fields×100)

Table S1:Lung injury scoring method based on histological parameters to quantify severity.

Table S2

| Antibodie | Companie&Catalog number | Dilution ratio |
| --- | --- | --- |
| ZO-1 Rabbit mAb | Cell Signaling Technology #13663 | 1:1000 |
| Occludin Rabbit mAb | Cell Signaling Technology #91131 | 1:1000 |
| Anti-IGF2BP1/IMP1（ZBP1）Rabbit mAb | Abcam ab290736 | 1:1000 |
| Anti-pro Caspase-1 + p10 + p12 Rabbit mAb | Abcam ab179515 | 1:1000 |
| Anti-GSDMD Rabbit mAb | Abcam ab209845 | 1:500 |
| Anti-NLRP3 Rabbit mAb | Abcam ab263899 | 1:500 |
| Cleaved Caspase-3 Rabbit mAb | Cell Signaling Technology #9664 | 1:1000 |
| Caspase-3 Rabbit mAb | Cell Signaling Technology #14220 | 1:500 |
| MLKL Rabbit mAb | Abclonal A19685 | 1:500 |
| Phospho-MLKL-S358 Rabbit pAb | Abclonal AP1244 | 1:500 |
| Caspase-8 Rabbit mAb | Cell Signaling Technology #4790 | 1:1000 |
| β-Actin (13E5) Rabbit mAb | Cell Signaling Technology #4970 | 1:1000 |

Table S2:The names, catalog numbers, and dilution ratios of the antibodies used in this study.
